# Levels of nicotine in Ethiopian tobacco leaves

**DOI:** 10.1186/s40064-015-1448-y

**Published:** 2015-10-29

**Authors:** Zebasil Tassew, Bhagwan Singh Chandravanshi

**Affiliations:** Quality Control Department, Ethiopian Pharmaceuticals Manufacturing Sh. Co. (Epharm), P.O. Box 2457, Addis Ababa, Ethiopia; Department of Chemistry, Bahir Dar University, P.O. Box 79 Bahir Dar, Ethiopia; Department of Chemistry, College of Natural Sciences, Addis Ababa University, P.O. Box 1176, Addis Ababa, Ethiopia

**Keywords:** Ethiopia, Tobacco leaves, Nicotine, High performance liquid chromatography

## Abstract

Tobacco is a valuable cash crop. It is the most widely grown non-food crop in the world. Tobacco use is widespread due to its addictive nature of its main constituent nicotine. Therefore, the knowledge of nicotine level in tobacco is important to tobacco industry and in the area of toxicology to control its harmful effect on health. There is no report in the literature on nicotine level of Ethiopian raw (unprocessed) tobacco leaves. Hence, the objective of this study is to determine the levels of nicotine in the Ethiopian tobacco leaves. Samples were collected based on their leaves positions, species and place of cultivation from different regions of Ethiopia. These were Virginia type tobacco from Shewa Robit and Billate, Burley and Oriental types of tobacco from Awassa and native tobacco used as pipe smoking (Gaya) from Wollayita. The level of nicotine in four different varieties of Ethiopian tobacco leaves was determined using high performance liquid chromatography. The level of nicotine in the four different varieties of Ethiopian tobacco were Virginia tobacco (3.26 %), the native tobacco ‘Gaya’ (1.10 %), Burley tobacco (0.650 %), and Oriental tobacco leaves (≤0.0500 %). It was found that the nicotine level of Ethiopian Virginia tobacco leaves increases from bottom to top leaf (stalk) positions of the tobacco plant. It was also found that the nicotine level of Ethiopian tobacco leaves varies in different species and the nicotine level of the same tobacco species differ in different area of cultivation. In general, the level of nicotine in Ethiopian tobacco is comparable with that in the rest of the world.

## Background

Tobacco is a valuable cash crop. It is the most widely grown non-food crop in the world with assistance of man and with the leaf as the most valuable part of the plant (Reed et al. 2012). Its growth is restricted by environmental factors. The majority of the tobaccos entering the world trade are produced in tropical and temperate regions, *i.e.* almost all continents are capable of growing tobacco, and hence many countries are tobacco producers. According to Food and Agriculture Organization (FAO) report in 2003; China, Brazil, India and United States are among the top ten leading countries to grow tobacco (FAO (Food and Agriculture Organization of the United Nations) 2003). Even though, the use of tobacco is detrimental to the user, the tobacco plant remains to this day one of the most important economic crops in the world. The reason tobacco use spread was the addictive nature of its main constituent nicotine which causes dependence (Benowitz 1996; Goedeker and Tiffany 2008).

Nicotine is principal tobacco alkaloid comprising about 95 % of total alkaloid content (Hukkanen et al. 2005; Vlase et al. 2005). Nicotine is pale yellow to dark brown oily liquid with unpleasant pungent odor, sharp persistent bitter taste and soluble in alcohol, chloroform, ether, etc. (Merck Index 2001; Hukkanen et al. 2005).

Nicotine is recognized as an essential component of tobacco cultivation since it determines the quality of tobacco in the tobacco market. In small doses nicotine has a stimulating effect, increasing activity, alertness and memory. But repeat users report only relief from the symptoms of nicotine withdrawal. It also increases the heart rate and blood pressure and reduces the appetite (Toustad and Andrew-Johnston 2006; Wang et al. 2009; Wu et al. 2009; Bastida and Beltran 2011). In large doses nicotine may cause vomiting and nausea. Large doses are poisonous to most animals and to humans. An aliquot of 40–60 mg can be a lethal dosage for adult human beings. This makes it an extremely deadly poison (Benowitz 1996; Saxena et al. 2003; International Labor Office 2012). It is more toxic than many other alkaloids such as cocaine (with lethal dose of 1000 mg) (Benowitz 1996; Saxena et al. 2003; International Labor Office 2012). Thus, nicotine level of tobacco should be quantitatively determined before their distribution in the market to minimize its risk.

Literature survey reveals that in Ethiopia peoples commonly use khat, tobacco, and alcohol, which have a share of 48.2, 29.9 and 18.9 %, respectively, of all type of drugs (Gebre Selassie and Gebre 1996). This shows that tobacco has a better place as a drug. Commonly Virginia, Oriental and Burley are commercial tobacco species used for making cigarettes in Ethiopia, besides these native tobacco species (Gaya) are used in the form of snuffing, chewing and pipe smoking.

Recently some studies have been carried out on the chemical composition of a psychoactive khat leaves (Atlabachew et al. 2010, 2011, 2013, 2014) as well as on the levels of essential and non-essential metals in cannabis leaves (Zerihun et al. 2015).

Determination of nicotine level in tobacco is crucial to control, maintain or reduce nicotine levels below the addictive as well as harmful threshold. Therefore, the knowledge of nicotine level in tobacco is especially important to tobacco industry and in the area of toxicology to control its harmful effect on health. There are two reports on the levels of nicotine in Ethiopian cigarette tobacco (Geto et al. 2012; Kassa et al. 2013). However, there is no report in the literature on nicotine level of Ethiopian raw tobacco leaves. Hence, it was worth to determine the levels of nicotine in some of Ethiopian raw tobacco leaves based on difference in species, cultivation areas and stalk (leaf) positions.

## Methods

### Equipments

High performance liquid chromatography system (HPLC 600 PerkinElmer, USA) with Totalchrom software consists of Series-200 Pump, Autosampler (automatic injector), UV/Vis detector, column oven and vacuum degasser, was used to determine the level of nicotine. The column used was Beckman Coulter C-18 reversed phase column (4.6 mm × 25 cm, USA). The UV–Vis spectrophotometer (CE4400/UV/Vis double beam scanning, USA), pH-meter (inoLab pH level 2, Weilhelm, Germany), drying oven (Heraeus Vacutherm, Japan), weighing balance (ACCULAB ALC-2104, USA), water bath with agitator (GFL, D-30938 Bergwedel, Germany) and sonicator (BANDELIN SONOREX RK514, Germany) were used.

### Chemicals

Standard nicotine 98 % (Riedel–de Haen, Germany), methanol HPLC grade (Fisher Scientific, UK), phosphoric acid and triethylamine (Riedel–de Haen, Germany) were used. A 25 mM phosphate buffer (pH 7.8) was prepared from potassium dihydrogen orthophosphate, KH_2_PO_4_ (Fisher Scientific, UK) and disodium hydrogen orthophosphate, Na_2_HPO_4_ (BDH Chemicals, UK) according to the procedure in British Pharmacopoeia (2012). All the experiments were done in The Chemical Laboratory of Ethiopian Pharmaceuticals Manufacturing Sh. Co. (EPHARM), Addis Ababa, Ethiopia.

### Nicotine solution

A 10 mM stock standard solution of nicotine was prepared in distilled water. Standard solutions of different concentrations (0.10, 0.50, 1.0, 1.5, 2.0, 2.5 and 3.0 mM) of nicotine were prepared by serial dilution with distilled water for the construction of calibration curve.

### Sample collection

Four types of tobacco leaves were collected from four different parts of the country. These were Virginia from Shewa Robit (250 km from Addis Ababa to the north) and Billate (about 330 km from Addis Ababa to the south), Burley and Oriental from Awassa (250 km from Addis Ababa to the south) and native tobacco (Gaya) from Wollayita (about 330 km from Addis Ababa to the south).

Virginia tobacco was collected from Shewa Robit at different leaf positions. The leaf positions were lugs, cutters, leaf and tips (from bottom to top). Although the time of harvesting (middle of October to middle of November) of particular farm site of the sample was different, cured samples of the tobacco leaf at the respective leaf position from the Shewa Robit site were also collected from the National Tobacco Enterprise for comparison of cured and sun-dry tobacco leaves. Thus a total of eight samples were analyzed from this site. Four samples of Virginia type tobacco was also collected from Billate at different leaf positions.

Burley and Oriental tobacco leaf, respectively, were collected from Kikea and Galley (7–10 km from Awassa). For these two species all the leaves from different positions of each species were dried, mixed and analyzed separately as two samples from this site. Since unlike that of Virginia type drying or curing style has no exaggerated effect on Burley and no effect on Oriental type of tobacco (Roemer et al. 2012).

Wollayita was the fourth sample site. Here there are many people who use native tobacco (Gaya) in different style; the most commonly used is pipe smoking. The leaves used for this purpose were collected as a one sample for analysis of its nicotine content.

Thus a total of 15 bulk samples were collected from the four selected sample sites.

### Nicotine determination

The determination of nicotine in the tobacco leaves was done by the method reported by Saunders and Blume (1981). Dried leaves of tobacco samples were ground in a mortar and a pestle, sieved with about 2 mm mesh, and oven-dried at 60 °C for 24 h to a constant weight. About 0.5 g lots weighed and extracted with 10 mL of 25 mM phosphate buffer (pH 7.8) in water bath adjusted at 30 °C for 24 h with constant agitation. The aqueous extract was filtered through filter paper and the filtrate was stored in refrigerator until injection to HPLC. The filtrate was diluted to ten-fold with water, sonicated for homogeneity and degassing of the sample (extract) solution. Finally, each extract was filtered through a 0.45 μm cellulose filter and sealed in a screw-capped septum vial to permit automatic injection of a 10 μL aliquot.

All quantitative determinations were made with triplicate injections. The extraction was done in triplicate for each leaf sample. The content of nicotine of each sample was then quantitatively determined based on the external calibration curve (Saunders and Blume 1981).

### Conditions used for HPLC

An isocratic mobile phase of 40 % (v/v) methanol containing 0.2 % phosphoric acid buffered to pH 7.25 with triethylamine and flow rate of 1 mL/min was used. The buffered mobile phase was prepared once for the whole analysis with great care by adjusting the intended pH value. Furthermore the mobile phase was used after filtration through a 0.45 μm cellulose filter and then 5 min sonication. The room temperature was used as a column temperature for this work.

UV/Vis detector was adjusted to detect nicotine at its maximum wavelength absorbance of 259 nm. Then the mobile phase was allowed to flow through the column or the line of flow of the instrument for some minutes until stable absorbance of the mobile phase observed. After observation of stable absorbance of the mobile phase using the instrument software it was set to out-zero, and the automatic injector was obeyed by the software program to inject automatically 10 µL of sample from the septum vials. As a result the nicotine peak was appeared at about 7.3 min at a flow rate of 1 mL/min.

The UV spectrum of 1 mM nicotine was recorded and the spectrum showed four λ_max_ of nicotine at 238, 254, 259 and 282 nm. Similar wavelengths have been reported in the literature (Clayton et al. 2013). To select the preferred λ_max_ of nicotine for the analysis 10 μL of 1 mM standard nicotine was injected to HPLC and the peak area at each of the above four wavelengths was recorded separately. The response peak area of nicotine at 259 nm was the largest. Hence 259 nm was selected for detection and quantification of nicotine. The chromatogram of standard nicotine was run at the optimum experimental conditions described above. The nicotine peak was appeared at about 7.3 min.

### Recovery test for method of extraction

The recovery test of the method of analysis used in this study was assessed by simultaneous extraction and determination of nicotine in the unspiked and spiked powdered tobacco leaves with 1.00 mM standard nicotine solution.

## Results and discussion

### Detection limit

The calibration curve was obtained by plotting the peak area response as a function of concentrations of nicotine in the standard solutions (0.1–3.0 mM) with its goodness of fit (r^2^) 0.9971. The equation of calibration curve is given by: y = 1,001,029x + 27,836, r^2^ = 0.9971, where y is the peak area response and x is nicotine concentration in mM. In this study the detection and quantification limits were determined by the definition given by International Union of Pure and Applied Chemistry (IUPAC) (Currie 1995). The detection limit (3σ_B_) and quantification limit (10σ_B_) were found to be 15 and 50 μM, respectively.

### Recovery test for method of extraction

The recovery of nicotine from the Virginia tobacco leaves was found to be 93–105 %. The results are given in Table 1. This result is in good agreement with the recovery percentages of 91–107 % reported by Geto et al. (2012) and 97–108 % reported by Kassa et al. (2013) by voltammetric methods.

**Table 1:** Data for recovery experiment^a^

Nicotine concentration (mM)			Recovery (%)
In unspiked tobacco leaves extract	Nicotine added	In spiked tobacco leaves extract	
1.56	1.00	2.50	93.4
1.50	1.00	2.43	93.0
1.44	1.00	2.49	105

^a^The recovery experiment was performed on the tip of Virginia tobacco leaf sample

### Nicotine levels of Shewa Robit and Billate Virginia tobacco leaves

Sun-dry tobacco leaves for this study mean that as soon as the leaves were collected, they were not exposed to direct sun light and dried. Instead, first they were allowed to pass through the yellowing process (gradual conversion of green to yellow leaf) by preventing the green leaf from direct sunlight. This yellowing process took almost 14 days, and then yellow leaves of tobacco were exposed to direct sun light to undergo gradual drying process. Finally dried leaves were obtained within a week sun light exposure. The effect of direct sun-drying on the nicotine content of tobacco leaves has been described elsewhere (Wang et al. 2008; Roemer et al. 2012).

The nicotine levels of sun-dry tobacco leaves of Shewa Robit and Billate Virginia tobacco are given in Table 2 based on the leaf positions lugs, cutters, leaf and tip with respective RSD for their triplicates. The results show that the nicotine level of tobacco leaves increases from bottom leaf position (lugs) to upper leaf position (tip) on both area of cultivation of Virginia tobacco. This is supported by literature (Hawks 1978). And their average nicotine levels were determined to be 2.20 and 3.26 %, respectively. The Billate Virginia tobacco showed the highest nicotine level of all the analysis results of this study.

**Table 2:** Comparison of nicotine levels of Virginia tobacco based on area of cultivation and leaf position

Stalk (leaf) position	Billate tobacco leaves (%)	Shewa Robit tobacco leaves (%)
Lugs	2.24 ± 0.07	1.75 ± 0.05
Cutter	2.48 ± 0.11	2.04 ± 0.06
Leaf	3.29 ± 0.18	2.18 ± 0.04
Tip	5.03 ± 0.27	2.81 ± 0.12
Mean	3.26 ± 0.16	2.20 ± 0.07

### Comparison of nicotine levels of Virginia tobacco leaves based on area of cultivation

Virginia tobacco leaves were collected from two samples sites Shewa Robit and Billate and their nicotine levels are presented in Table 2 with their respective leaf positions.

The nicotine level of Billate tobacco leaves is higher than that of Shewa Robit tobacco leaves. This is shown by the differences in the average nicotine level of the whole leaf as well as nicotine levels of different leaf positions. This may be accounted by many factors. The main factor is use of different amount of fertilizers. Furthermore, the cultivation area of tobacco in Billate is greater than that of Shewa Robit. Billate is state owned while in Shewa Robit individual farmers own the cultivation areas. The extent of use of fertilizers by the state and individual farmers are different. Moreover atmospheric conditions such as temperature, rainfall, humidity of the cultivation area, cultural practice of cultivation and maturity level contribute significant difference on nicotine levels of the tobacco leaves of the two places.

### Nicotine levels of Awassa Burely and Oriental tobacco leaves

From Awassa sample site Burley and Oriental tobacco species were collected. The nicotine level of Burley tobacco was found to be 0.650 %. While for Oriental tobacco leaves it was difficult to found reliable value of nicotine level, it was not possible to get well-integrated nicotine peak area by this method of analysis and set of conditions.

### Nicotine level of Wollayita native tobacco leaves ‘Gaya’

The nicotine level of ‘Gaya’ of Wollayita, which is one of the well known native tobacco species in Ethiopia was found to be 1.11 %, which is almost fair level of nicotine as compared with other tobacco species, especially with Virginia.

### Comparison of nicotine level of Ethiopian tobacco leaves based on their species

The nicotine level of Billate Virginia tobacco leaves is high as compared with others, followed by Shewa Robit Virginia tobacco leaves. Native tobacco leaves ‘Gaya’ possess middle place of nicotine level, and then Burley tobacco leaves contain very low nicotine. The nicotine level in Oriental tobacco leaves was roughly equal to the detection limit and its’ accurate level of nicotine was not determined by this study. The chromatogram of Oriental tobacco leaves shows that the presence of nicotine in trace level, since very small non-integrated peak of nicotine was observed in the chromatogram of oriental tobacco leaves. The results are given in Table 3.

**Table 3:** Comparison of nicotine levels (%) based on their species

Type of species of tobacco	Average nicotine level (%)
Virginia (Billate)	3.26 ± 0.16
Virginia (Shewa Robit)	2.20 ± 0.07
Burley (Awassa)	0.650 ± 0.02
Oriental (Awassa)	ND (≤0.050)
Gaya (Wollayita)	1.11 ± 0.05

*ND* not detected. It was roughly equal to detection limit

### Comparison of nicotine level of Ethiopian tobacco leaves with literature data from other countries

The nicotine content of tobacco leaves generally ranges from 0.3 to 3 %, though 5 % and even 7 % have been recorded in some heavy bodied tobaccos (Blakely and Bates 1998). Wang et al. (2008) in a study from China reported the nicotine level ranging from 0.78 to 3.26 % depending upon different leaf position and different treatment. Geto et al. (2012) reported the nicotine level in two brands of Ethiopian cigarette tobacco as 3.84 and 4.26 %, while Kassa et al. (2013) reported the nicotine levels in another two brands of Ethiopian cigarette tobacco as 2.04 and 2.54 %. Thus, the nicotine level of Ethiopian raw (unprocessed) Virginia tobacco leaves (3.26 %) are comparable to the nicotine levels reported in other studies. While the nicotine levels in other two Ethiopian raw tobacco leaves (Gaya 1.11 % and Burley 0.650 %) are lower than that in Virginia but comparable to that in the other tobacco leaves (Blakely and Bates 1998; Wang et al. 2008).

## Conclusion

The level of nicotine in the Ethiopian tobacco leaves were determined by high performance liquid chromatography. The analysis of tobacco leaves was based on their leaf positions, places of cultivation and variety of species. Among the variety of species Virginia tobacco leaves were found to have high nicotine level (3.26 %), followed by the native tobacco ‘Gaya’ (1.11 %) then Burley tobacco leaves (0.650 %). The nicotine level in Oriental tobacco leaves was below the detection limit and its’ accurate level of nicotine was not determined by this study. Based on their leaf positions, the nicotine level of Ethiopian Virginia tobacco leaves like that of other countries increases from bottom to top (tip) leaves. Furthermore this study has found that nicotine level of a given tobacco species cultivated in different places is different. The levels of nicotine in Ethiopian tobacco leaves are comparable with that cultivated in the rest of world (Blakely and Bates 1998; Wang et al. 2008; Geto et al. 2012; Kassa et al. 2013).
